# TCF4 at the crest of development: a zebrafish model to explore craniofacial and gastrointestinal defects in Pitt-Hopkins syndrome

**DOI:** 10.3389/fcell.2026.1907674

**Published:** 2026-09-04

**Authors:** Martina Orefice, Francesca Remondino, Irene Pieraccioni, Giulia Salamone, Silvia Savoli, Antonio Vitobello, Chiara Gabellini, Michela Ori

**Affiliations:** 1 Department of Biology, Unit of Cellular, Molecular and Developmental Biology, University of Pisa, Pisa, Italy; 2 Université Bourgogne Europe, CHU Dijon Bourgogne, Laboratoire de Génomique Médicale, Centre Neomics, FHU-TRANSLAD, Centre de Recherche Translationnelle en Médecine Moléculaire - Inserm UMR1231, équipe GAD, Dijon, France

**Keywords:** CRISPR/Cas9, enteric nervous system, neural crest cells (NCCs), PHOX2B, pitt-hopkins syndrome, rare disease, zebrafish

## Abstract

**Background:**

Pitt-Hopkins Syndrome (PTHS) is a rare neurodevelopmental disorder caused by haploinsufficiency of the *TCF4* gene. It is characterized by intellectual disability, distinctive facial features, breathing abnormalities, and gastrointestinal dysfunction. While the role of TCF4 in central nervous system development has been extensively investigated, the developmental basis underlying craniofacial and enteric alterations remains poorly understood.

**Methods:**

We generated a zebrafish *tcf4* mutant line using CRISPR/Cas9 genome editing to unveil the role of Tcf4 in neural crest-derived lineages that contribute to craniofacial and Enteric Nervous System development. The model was characterized by multiple integrated approaches to evaluate the morphological, cellular and functional alterations associated with *tcf4* haploinsufficiency. As a proof-of-concept that the observed phenotypes resulted from Tcf4 loss of function, we reinstated human TCF4 expression in mutant larvae and assessed the rescue of the pathological phenotypes previously described.

**Results:**

Heterozygous mutants exhibit key features of PTHS, including craniofacial skeletal abnormalities and impaired gastrointestinal motility. Functional analysis revealed a significant reduction of spontaneous peristaltic contractions and a delayed swallow-induced gut transit, consistently with human patients and PTHS mouse model. To further elucidate these developmental alterations, we showed that these defects are associated with a reduced number of Phox2b-positive enteric progenitors and HuC-positive enteric neurons, though early vagal neural crest migration appears unaffected. Reintroducing human TCF4 mRNA in tcf4 heterozygous mutant embryos rescues both craniofacial and gastrointestinal phenotypes, confirming the specificity of the observed phenotypes. Furthermore, we showed that the human *TCF4* mRNA overexpression can alter craniofacial development in zebrafish embryos suggesting that TCF4 reinstatement dosage should be evaluated in gene therapy approaches to avoid a gain of function phenotype.

**Conclusion:**

Together, our results shed light on TCF4 as a key regulator of neural crest-derived lineages and provide a new perspective on two major clinical features of PTHS. The *tcf4* mutant line represents a novel in vivo platform for investigating PTHS pathogenesis at cellular and molecular level and testing novel therapeutic strategies

## 1 Introduction

Pitt-Hopkins Syndrome (PTHS) is a rare neurodevelopmental disorder first described in 1978 (Pitt and Hopkins, 1978), characterized by developmental delay, distinctive craniofacial features, intellectual disability, impaired motor coordination, hyperventilation, lack of speech, and gastrointestinal dysfunction. In 2007, *de novo* heterozygous pathogenic variants in *TCF4* were identified as the primary cause of PTHS, leading to *TCF4* haploinsufficiency (Amiel et al., 2007; Zweier et al., 2007). Transcription Factor 4 (TCF4, OMIM: 602272) is a class I basic helix-loop-helix (bHLH) E-protein that plays a crucial role in early brain development. The bHLH domain mediates both homodimerization and heterodimerization, enabling TCF4 to bind to specific DNA sequences and regulate gene expression programs involved in the Central Nervous System (CNS) development and function. Notably, this domain represents a mutational hotspot in PTHS patients, with pathogenic missense variants clustering in this region (Whalen et al., 2012).

Variants with loss-of-function (LoF) effect have been shown to impair early brain development both *in vitro* and *in vivo* models (Brockschmidt et al., 2007; Bedeschi et al., 2017; Chen et al., 2021). CNS-specific homozygous *Tcf4* knock-out mice survive after birth and exhibit microcephaly and delayed neuronal differentiation (Quednow et al., 2014; Schoof et al., 2020). This is consistent with behavioural alterations in mice, such as hyperactivity, decreased anxiety and impaired spatial learning and memory (Thaxton et al., 2018). Recent studies on PTHS patient-derived neural progenitors have revealed reduced proliferation, impaired neuronal differentiation, and decreased WNT signaling and SOX genes expression (Papes et al., 2022; Espinoza et al., 2024).

Despite several studies have demonstrated the crucial role of TCF4 in neurodevelopmental processes, the molecular and pathophysiological mechanisms underlying some of the PTHS patients’ clinical features remain poorly understood. Those include the developmental origin of the facial gestalt, currently recognized as one of the principal diagnostic criteria for PTHS (Zollino et al., 2019), and of the gastrointestinal (GI) complications, such as constipation (80% in children, and 70% in adults), which could persist lifelong and is often severe. Reduced bowel transit leads to intestinal pseudo-obstruction and abdominal distension, resulting in persistent patient agitation (Comisi et al., 2023). These defects have been hypothesized to arise from dysfunction of the Enteric Nervous System (ENS) (Zollino et al., 2019), although direct evidence is still limited.

Whether the craniofacial and gastrointestinal manifestations of PTHS share a common developmental origin remains unknown. Notably, the embryonic cartilaginous craniofacial skeleton derives from the cranial Neural Crest Cells (cNCC) while the Enteric Nervous System derives from a subpopulation of the trunk NCC, the enteric Neural Crest Cells (eNCC). Despite this shared developmental origin, the potential role of TCF4 in NCC biology has not been investigated so far. Our research aims to address this knowledge gap by exploiting zebrafish embryos as a novel *in vivo* model of PTHS, enabling direct visualization of Neural Crest Cells (NCC) derivatives development. This approach provides new insights into the contribution of TCF4 to NCC-derived lineages and offers a platform for studying the developmental basis of PTHS-associated phenotypes. Improving our understanding of understudied aspects of PTHS could significantly impact on the quality of life of patients and their families, making this area of research of major significance for the development and validation of targeted interventions.

## 2 Materials and methods

### 2.1 Animal care and husbandry

Adult zebrafish male and female (wild type AB strain) were housed according to standard procedures on a 14-h light/10-h dark cycle (Aleström et al., 2020). Zebrafish embryos and larvae were collected and raised at 28.5 °C in E3 embryo medium and staged in hours post fertilization (hpf) or days post fertilization (dpf). Every animal procedure was performed following protocols approved by Italian Ministry of Public Health and the local Ethical Committee of the University of Pisa (authorization n. 693/2025-PR) in conformity with the European directive 2010/63/EU and following the 3R principles (MacArthur Clark, 2018).

### 2.2 Generation of a *tcf4* mutant line in zebrafish

A CRISPR/Cas9-based approach was used to generate a stable *tcf4* mutant line. The crRNA targeting zebrafish *tcf4* locus (ENSDARG00000107408) was designed using CHOP-CHOP software (https://chopchop.cbu.uib.no/) (danRer11/GRCz11 assembly). The designed crRNA (20 nucleotide complementary to the target: CAA​ACT​GCT​GAT​CCT​GCA​TC), SpCas9 tracrRNA and Alt-R S. p.HiFi Cas9 nuclease V3 were bought by Integrated DNA Technologies (IDT). Following the manufacturer’s instructions, the crRNA specific for *tcf4* locus was annealed with the universal tracrRNA (1:1 ratio) and complexed with spCas9 (1:1 ratio) to obtain the RNP complex. Gene editing in mosaic embryos at 48 hpf (hours post fertilization) was confirmed by High Resolution Melting analysis (HRM), with SensiFast™ HRM kit (Bioline), following manufacturer’s instructions, in the QuantStudio 3 real-time PCR system (AppliedBiosystems). When HRM confirmed successful gene editing, the remaining F0 embryos were bred to adults in the husbandry. The F0 adults were then outcrossed with wild-type individuals to identify a founder able to transmit *tcf4* gene mutations through the germline. F1 progeny was screened by HRM analysis and successful editing was confirmed by Sanger sequencing. Presumptive off-targets were analysed using Cas-OFFinder (CRISPR Rgen Tool, http://www.rgenome.net/cas-offinder/) and the modification of genes including a similar RNA guide target up to 4 mismatches was excluded by Sanger sequencing. The relative expression of *tcf4* was evaluated by RT-qPCR. Total RNA was extracted by a pool of 30 embryos at 3 days post fertilization (dpf), using the *RNeasy® Plus Mini Kit* (Qiagen), with DNAse I treatment. Reverse transcription was performed by *QuantiTect Reverse Transcription Kit* (Qiagen), following manufacturer’s instruction. qPCR was performed using *GoTaq® qPCR Master Mix* (Promega). The reference gene used was *eif1a*. Primers used are listed in Table 1.

**TABLE 1:** List of primers used for the tcf4 knock-out line in zebrafish.

Name	Sequence (5’ > 3′)	%Gc	Tm (°C)
tcf4_HRM_F	CGTGTGCGCGACATCAA	17	66
tcf4_HRM_R	GCT​GCT​CCA​GGC​TGA​GTA​T	19	66
tcf4_F_PCR	TGA​TGA​GCA​GGA​CCT​GAC​GC	20	61.4
tcf4_R_PCR	CCC​ATG​GGA​TTG​GAC​GCC​T	19	61
tcf4_F_seq	TGA​TGA​CGA​GGA​CCT​GAC​G	19	58.8
tcf4_R_seq	ATGGGATTGGACGCCTC	17	55.2
eif1a_F_qPCR	AGA​AGA​TCG​ACC​GTC​GTT​CTG	21	60
eif1a_R_qPCR	CTC​ATG​TCA​CGC​ACA​GCA​AAG	21	60
tcf4_F_qPCR	TGA​CAG​TAA​TGG​AGA​CCT​GAC​G	22	60.3
tcf4_R_qPCR	CTC​CTT​GAA​GGC​CTC​GTT​GAT	21	59.8

### 2.3 Genotyping

Genomic DNA was extracted from larvae fins (fin clipping) at 3 dpf by proteinase K (EO0492, ThermoFisher Scientific) and amplified by PCR using GoTaq® G2 Hot Start Green Master Mix (M7422, Promega) with GeneAmp® PCR System 9,700 (Applied Biosystems), according to manufacturer’s instructions. PCR products were purified using Wizard® SV Gel and PCR Clean-Up System (A9281, Promega) and the concentration of total DNA was determined by NanoDrop™. Sequence data were obtained by Sanger method (Eurofins Scientific, Germany) and analysed with different tools, CRISP-ID software (http://crispid.gbiomed.kuleuven.be/) (Dehairs et al., 2016) and Poly Peak Parser software (http://yosttools.genetics.utah.edu/PolyPeakParser/) (Hill et al., 2014) to identify sequence alterations in *tcf4* mutants. The specific deletion identified in the male chosen as founder was then genotyped in the next generations by *TaqMan SNP Genotyping Assays Work*, designed by Thermo Fisher Scientific (assay ID: AN2XVGW). Primers used are listed in Table 1.

### 2.4 Cartilage staining

Craniofacial structures of zebrafish embryos were stained with Alcian blue to highlight their architecture. Zebrafish embryos at 5 dpf were fixed in 4% paraformaldehyde (PFA) in phosphate buffered saline (PBS) at room temperature (RT) for 1–2 h. After washing in PBT, embryos were bleached with 1% KOH and 3% H_2_O_2_ for 30 min. Alcian Blue stain was dissolved by 1% HCl and 70% EtOH at 0.1 g/100 mL. The embryos were incubated with staining solution overnight and then washed for 4 h with 70% EtOH and 5% HCl. After staining, cranial cartilages were dissected using fine forceps. The structures of branchial arches and ethmoidal plates were examined in stained embryos. Images of embryos were obtained by the stereomicroscope Nikon SMZ18. Meckel’s-Meckel’s and PQ-Meckel’s angle were calculated by Fiji software (Schindelin et al., 2012) as previously described (Huang et al., 2021).

### 2.5 Whole mount *in situ* hybridization

The antisense probe specific for *tcf4* and *ret* transcript were generated by amplification of wild-type zebrafish cDNA (*tcf4*, For 5′-TCA​GTC​CAG​AGA​CGG​TTT​CTC-3′, Rev 5′-ACC​ATG​AGC​GAG​TGT​CTG​TTG-3′; *ret*, For 5′-AGC​TGG​ATA​CAC​TAC​GGT​TGC-3′, Rev 5′- CCG​CAA​GAT​CCA​GAT​AGT​CTC-3′) and cloning in p-GEM®-T vector (Promega). The digoxigenated sense (as negative control) and antisense probe were *in vitro* transcribed with T7 and SP6 RNA polymerase respectively (Promega) and digoxigenin–NTP mixture (Dig-NTP mix, Roche). Zebrafish embryos at specific developmental stages were collected and fixed in 4% PFA to preserve tissue structure and mRNA integrity. After verifying the probes’ specificity by sense probe hybridization, the whole-mount ISH was performed as previously described (Masson et al., 2025).

### 2.6 Immunofluorescence

Zebrafish embryos at 3 and 5 dpf developmental stages were collected and fixed in 4% PFA. Immunofluorescence was performed as previously described (Santos et al., 2018). To reduce the background in the intestinal lumen 0.1% Tween-20 was substituted with 0.3%/0.5% Triton X-100 in all the passages requiring PBTw. The permeabilization was performed in ice with PBTriton 2% for 30 min. The primary antibody used are: HuC/HuD (16A11. Invitrogen, 1:500), Phox2b (SC-376997, Santa Cruz, 1:1,000). Images of embryos were obtained by the stereomicroscope Nikon SMZ18. Embryos were mounted on slides with Fluoromount™ Aqueous Mounting Medium (Sigma-Aldrich) and examined with confocal microscope Nikon Eclipse Ti2, with a 14 µm depth of focus to discriminate single cells. Images were analysed using Fiji software.

### 2.7 Functional analysis

The live imaging of peristaltic movement of the gut was performed in *tcf4* +/− and *tcf4 +/+* zebrafish larvae. Larvae were kept 40 min in E3 medium with Alizarin Red S (A5533, Sigma-Aldrich) 0.01% at 28 °C. The ingestion of the medium by the larvae results in the emission of fluorescence by the Alizarin Red in the gut, allowing the live imaging of peristaltic movement, performed at the stereomicroscope Nikon SMZ18. After the treatment the larvae were washed with fresh medium and included in low gelling agarose (A9414, Sigma-Aldrich) 0.08% with 1X tricaine methanesulfonate. Video recording was performed at 100 fps (frame per second) for 4 min and saved with AVI format. Video analysis was performed with MATLAB software (https://it.mathworks.com/products/matlab.html?s_tid=m_footer_matlab). The scripts for the analysis were specifically designed to obtain a matrix describing the variation of pixel intensity over space and time during the recording, which reflects the underlying peristaltic movement (Supplementary material 2). The matrix was filtered to remove imaging artifacts and used to generate a kymograph of pixel movement showing time (s) in function of space (μm). The kymograph was then used to extrapolate information such as propagation and contraction velocities, and frequency of the peristaltic wave. The gut transit analysis was performed in zebrafish larvae following the procedures described (Rabahi et al., 2026). The day of registration larvae were fed with dry food. After 1 h from 8 to 10 larvae for each genotype were selected based on the presence and the position of the bolus in the gut, and isolated in fresh egg medium. Larvae were kept at 28 °C in the incubator. At 3, 6, and 24 h post feeding each larva was anesthetised with 1X tricaine methanesulfonate (MS-222, Sigma-Aldrich) and photographed with the stereomicroscope Nikon SMZ18. Images were analysed by Fiji software to evaluate bolus position. In particular, the gut was divided into three zones, based on somite’s borders, and the presence of bolus was counted in the zones for each embryo.

### 2.8 Functional rescue experiments

The coding sequence of human *TCF4* (NM_001083962) from OriGene (RC224345) was subcloned in pCS2+ vector. The *in vitro* transcription of *TCF4* mRNA was performed with mMessage mMachine SP6 Transcription Kit (Invitrogen). The transcript was diluted at 500 ng/μL and conserved at −80 °C. *TCF4* transcript was microinjected in zygote stage embryos into the yolk of *tcf4 +/+* and *tcf4* +/− at a final concentration of 200 pg per embryo for rescue experiment. The embryos were then raised to 5 dpf and fixed in 4% PFA. Only the embryos that met predefined inclusion criteria, such as successful injection and verified by co-injection of *gfp* mRNA, were used for downstream analysis, leading to different sample sizes. Cartilage staining, gut transit, immunofluorescence, imaging and cell counting was performed as previously described.

### 2.9 Statistical analysis

Statistical analysis was performed using GraphPad Prism version 9.0 (GraphPad Software, San Diego, CA, United States). Continuous variables were expressed as mean ± standard error of the mean (SEM), while categorical variable were presented as frequencies or percentages. Normality was assessed using Shapiro-Wilk test. Comparisons between two groups were performed using Student’s t-test or the Mann-Whitney U test, depending on the data distribution. Categorical variables were analysed using Fisher’s exact test or Chi-square test. Comparisons among more than two groups were performed using one-way ANOVA or the Kruskal–Wallis test, which accommodates moderate differences in sample sizes, making it suitable for the statistical comparisons of functional rescue experiments. Appropriate *post hoc* tests were applied, when applicable. The chosen level of significance was α = 0.05.

### 2.10 Use of AI-tool

AI model called Gemini 3.1 was used to develop a script for analysing gut peristalsis activity videos using MATLAB software. The model was used to assist with writing the code, verifying that it functioned correctly, and ensuring the accuracy of the output data.

## 3 Results

### 3.1 Zebrafish *tcf4* is dynamically expressed during embryonic development

**FIGURE 1:**
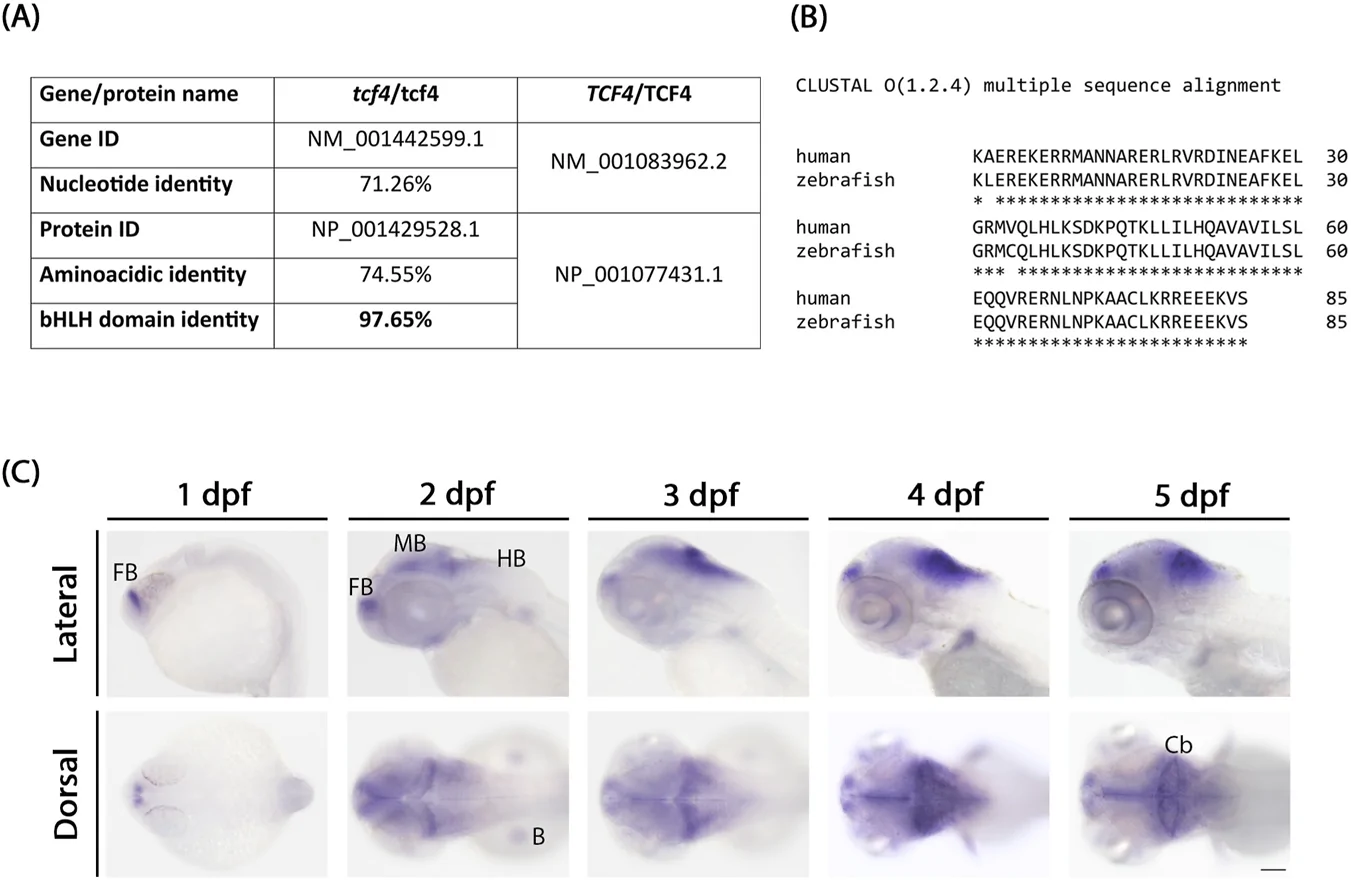
**(A)** Identity percentage obtained by BLAST alignment of zebrafish and human TCF4 transcript and protein, both for the total sequence and the bHLH domain. **(B)** Clustal Omega alignment of bHLH domain protein sequence between human and zebrafish TCF4. **(C)** Expression pattern of *tcf4* during zebrafish development. From right to left, at 1 dpf (days post fertilization) *tcf4* is expressed in the forebrain (FB). Later, at 2 and 3 dpf its expression expands to midbrain (MB) and hindbrain (HB), maintaining a high expression in the ventricular zone and in the midbrain-hindbrain boundary. The expression at these stages is also present in pharyngeal arches (PA) and fin bud **(B)**. At 4 dpf *tcf4* expression is strong in the hindbrain (HB), and its expression is maintained in the ventricular zone, the forebrain (FB), the pharyngeal arches (PA) and fin bud. At 5 dpf *tcf4* expression is strong in the cerebellum (Cb), forebrain (FB) and ventricular zone. Scale bar is 100 µm.

To assess the evolutionary conservation of TCF4, we aligned by BLASTp and Clustal O alignment tools the human (NP_001077431.1) and the zebrafish (NP_001429528.1) protein sequences, revealing high degree of conservation within the bHLH domain (97.65% identity), supporting functional conservation across vertebrates (Figures 1A,B). We next examined *tcf4* expression during zebrafish embryogenesis by whole-mount *in situ* hybridization (Figure 1C). Coherently with other vertebrates, *tcf4* is strongly expressed in the developing brain of zebrafish embryos, including the forebrain at 1 day post fertilization (dpf) and its distribution expands to midbrain and hindbrain regions moving forward in development. From 2 dpf onward, *tcf4* expression was also observed in pharyngeal arches, which give rise to craniofacial structures, and in the hindbrain-midbrain boundaries, which regulates the patterning of the midbrain and the cerebellum. From 3 to 5 dpf its expression became more restricted to the cerebellum and hindbrain. This result suggests that *tcf4* is dynamically expressed in both CNS regions and in NCC-derived structures during vertebrate development.

### 3.2 *tcf4* knock-out in zebrafish models Pitt-Hopkins syndrome

**FIGURE 2:**
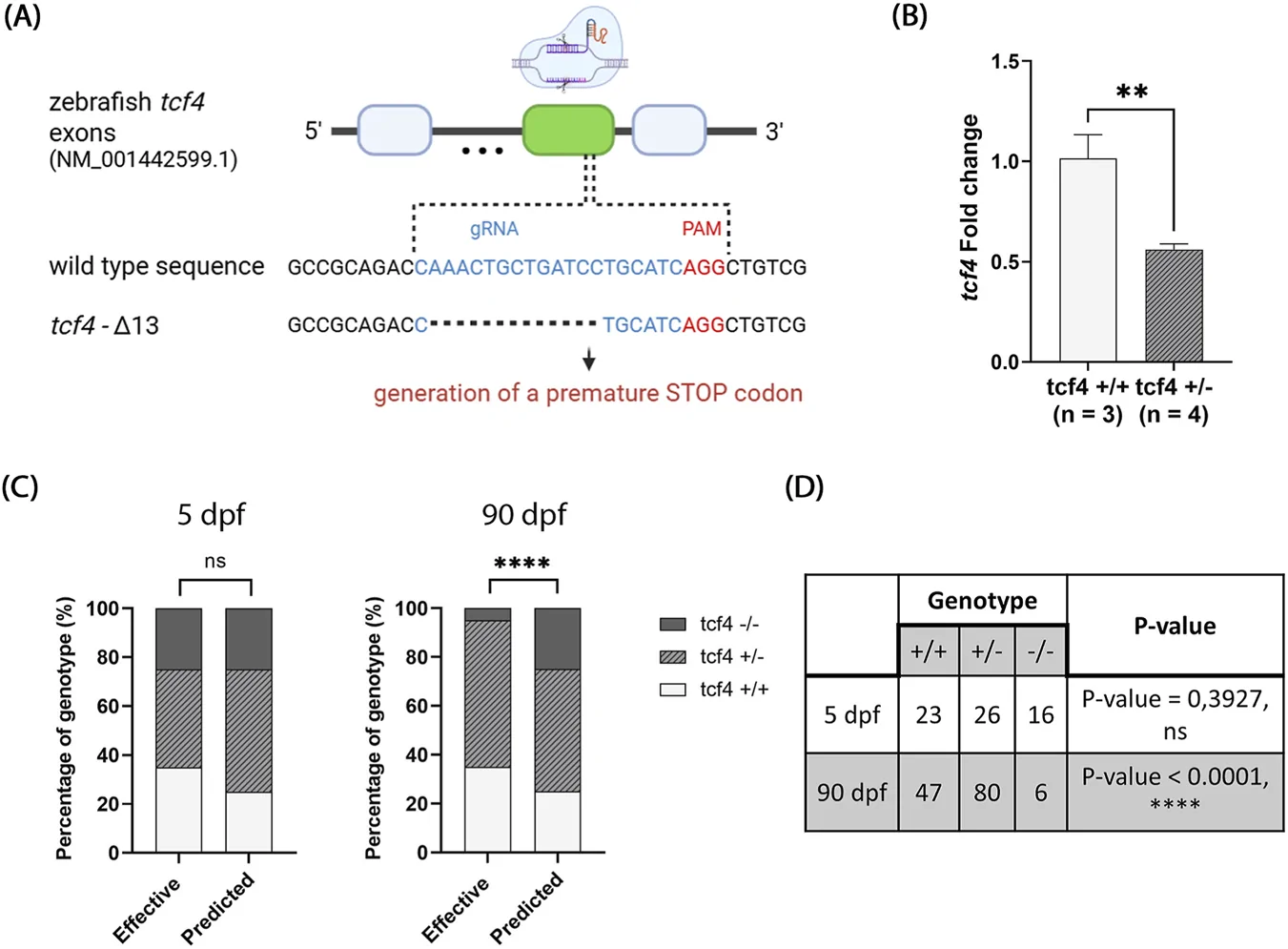
**(A)** Schematic representation of the experimental design for the CRISPR/Cas9 genome editing on *tcf4* locus. The sgRNA was designed to target *tcf4* exon coding for the bHLH domain, which represents a mutational hotspot for the PTHS. The CRISPR/Cas9 complex induced a 13 bp deletion, determining the formation of a premature STOP codon. Created with BioRender.com. **(B)** *tcf4* relative expression in *tcf4* +/− embryos is significantly reduced compared with *tcf4 +/+* embryos. Unpaired t-test, data are shown as *mean* ± *SEM.* **(C)** Graphical representation of genotype proportions observed and predicted in juvenile and adult fish and the relative significance. **(D)** Table of zebrafish juvenile (5 dpf) and adult (90 dpf) derived from mating between *tcf4* +/− fishes showing the number of individuals for every genotype, and the p-value obtained comparing the proportions observed with Mendelian proportions; Chi-square test.

We generated a stable mutant line for *tcf4* in zebrafish using CRISR/Cas9 targeting exon 17 (ENSDARE00001301438), which encodes for the bHLH domain. The RNP complex was injected in the zebrafish zygote. F0 injected embryos were screened by High Resolution Melting (HRM) analysis to evaluate the genome editing event, while the remaining mosaic embryos were raised to adulthood. Once adult, the F0 individuals were outcrossed with wild-type fish, and F1 embryos were screened by HRM analysis to identify the F0 fish ability to transmit mutations to their progeny. Among the identified alleles, we selected a 13-nucleotide deletion resulting in a frameshift and a premature stop codon within the region encoding the bHLH domain, predicted to cause loss-of-function (Figure 2A). Quantitative RT-PCR analysis at 3 dpf showed a significant reduction of *tcf4* transcript levels in *tcf4* +/− compared with *tcf4 +/+* embryos (unpaired t-test, p-value = 0.0077, **). Noteworthy, heterozygous embryos’ transcript levels were reduced to approximately half of those detected in wild-type siblings (n = 4, mean = 0.56, SEM = 0.02858 vs. n = 3, mean = 1.013, SEM = 0.1192), suggesting activation of nonsense-mediated decay (Figure 2B). Importantly, this reduction mimics the *TCF4* haploinsufficiency observed in patients with Pitt-Hopkins Syndrome, further validating this mutant line as a disease model for the syndrome.

In mice, homozygous null *Tcf4* mutants die before birth or at postnatal day 1 (P1), suggesting a fundamental role of TCF4 during development (Thaxton et al., 2018). To evaluate the impact of *tcf4* loss on zebrafish viability, we analysed the genotype distribution in offspring derived from heterozygous crosses. At 5 dpf, genotype frequencies followed Mendelian expectations. However, in adulthood (90 dpf), homozygous mutants were significantly underrepresented (Figures 2C,D; n = 133, Chi-square test, p-value<0.0001, ****), indicating reduced survival. These data highlight a fundamental and evolutionary conserved role of *tcf4* in vertebrate development.

**FIGURE 3:**
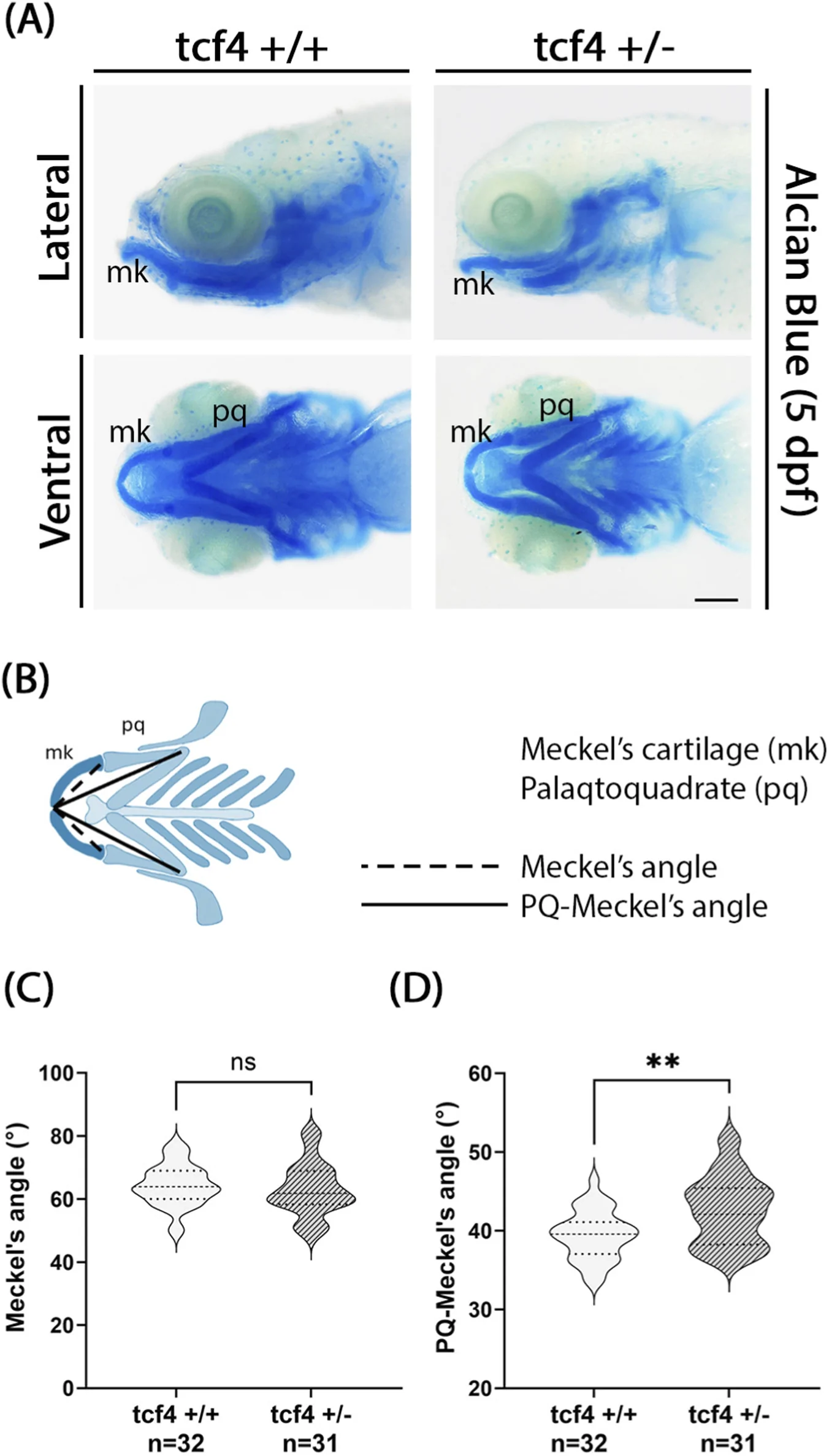
**(A)** Craniofacial skeletal developmental alterations observed in *tcf4* +/− larvae, both in lateral and ventral view, particularly affecting the Meckel’s cartilage (mk) and palatoquadrate (pq) angles. **(B)** The schematic representation of the craniofacial structures of zebrafish indicates the angles measured, namely, Meckel’s angle (dashed lines) and the PQ-Meckel’s angle (solid line). **(C,D)** Statistical analysis of the Meckel’s angle and PQ-Meckel’s angle measurement, with number of statistical unit and relative level of significance; Unpaired t-test. Scale bar is 100 µm.

Given that craniofacial gestalt is a hallmark of PTHS, we assessed craniofacial cartilage development in *tcf4* +/− larvae at 5 dpf using Alcian Blue staining (Figure 3A). Morphological analysis revealed alterations in structures derived from the first pharyngeal arch (Figure 3B). In particular, the palatoquadrate-Meckel’s (PQ-Meckel’s) angle was significantly (Figure 3C; unpaired t-test with Welch’s correction, p-value = 0.0016, **) increased in *tcf4* +/− larvae (n = 31, mean = 42.60, SEM = 0.7770) compared with *tcf4 +/+* larvae (n = 32, mean = 39.44, SEM = 0.5503), suggesting a widening of the jaw, akin to the wide mouth phenotypic trait observed in PTHS patients. In contrast, the Meckel’s angle was not significantly affected (Figure 3D; unpaired t-test, p-value = 0.4588, ns). Despite anatomical differences between zebrafish and mammals, the craniofacial structures of vertebrates develop from the chondrification of NCC-derived cartilaginous primordia, and the developmental processes driving their specification and morphogenesis are highly conserved. Thus, these findings support the hypothesis of a role of *tcf4* in craniofacial development.

### 3.3 *tcf4* +/− zebrafish larvae showed an impaired gut functionality

**FIGURE 4:**
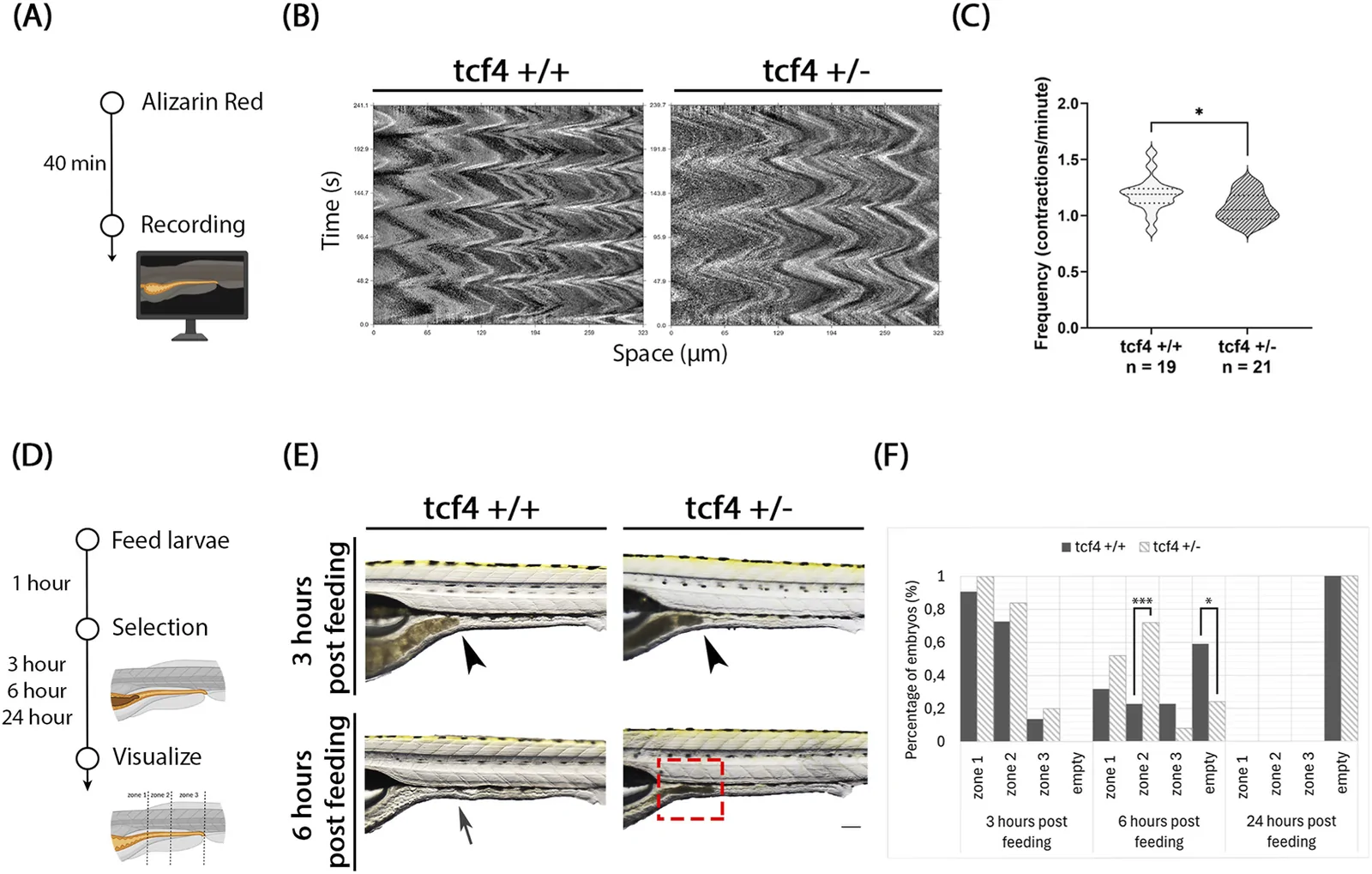
**(A)** Schematic representation of spontaneous basal peristaltic waves quantification workflow. Created with BioRender.com. **(B)** Kymograph obtained by the analysis of live-imaging videos of fasted *tcf4 +/+* and *tcf4* +/− larvae. **(C)** The frequency of peristaltic waves in *tcf4* +/− larvae was significantly reduced compared with *tcf4 +/+* larvae. Unpaired t-test. **(D)** Schematic representation of gut transit workflow. Created with BioRender.com. **(E)** Representative images of gut transit analysis. At 3 h post feeding both the conditions show the presence of the bolus (arrowhead). However, at 6 h post feeding, while most of the *tcf4 +/+* had successfully thrived (arrow), *tcf4* +/− displayed a delayed gut transit (dashed red box). Scale bare is 100 µm. **(F)** The percentage of *tcf4* +/− zebrafish larvae that emptied at 6 h post feeding was significantly reduced compared to *tcf4* +/+ embryos. The gut resulted completely empty at 24 h post feeding. *Tcf4 +/+* n = 22, *tcf4* +/− n = 25, Chi-square test.

Patients with PTHS present gastrointestinal (GI) complications, frequently including severe chronic constipation (75%). Zebrafish have been shown to be a good model for studying gastrointestinal diseases due to their small size and transparency of the larvae (Doodnath et al., 2010), which allow *in vivo* live imaging. To determine whether heterozygous *tcf4* loss affects gastrointestinal function, we analysed both spontaneous gut motility and swallow-induced gut transit in larval zebrafish. The quantitative analysis of spontaneous basal peristaltic movement was performed in fasted larvae (Figure 4A). The video was analysed with MATLAB software, obtaining spatiotemporal maps to extrapolate various parameters, including frequency of peristaltic waves, measured as contractions per minute. This analysis revealed a significant reduction (Figures 4B,C; unpaired t-test, p-value = 0.0180, *) in contraction frequency in *tcf4* +/− larvae (n = 21, mean = 1.077, SEM = 0.02781) compared with *tcf4 +/+* (n = 19, mean = 1.187, SEM = 0.03512), suggesting an impaired ability to generate effective peristaltic waves. To evaluate whether this phenotype could translate into an altered function, we assessed swallow-induced gut transit on *tcf4* +/− larvae. Larvae were fed and monitored over time for progression of the food bolus along the intestine (Figure 4D). At 6 h post feeding, *tcf4 +/−* (n = 25) larvae showed a significant delay (Figures 4E,F; Chi-square test, p-value = 0.0144, *) in gut transit compared to *tcf4 +/+* (n = 22), with a retention of the bolus in the rostral intestinal region (Figure 4F; Chi-square test, p-value = 0.0007, ***). By 24 h post feeding, all larvae had cleared the gut, indicating a delayed rather than a completely impaired motility, consistent with PTHS-related gastrointestinal complications. Together, these results are in line with altered gut function observed in Pitt-Hopkins mouse models and in human patients, supporting that *tcf4* haploinsufficiency leads to functional impairment of gastrointestinal motility.

### 3.4 *tcf4* loss of function compromises the enteric nervous system development

**FIGURE 5:**
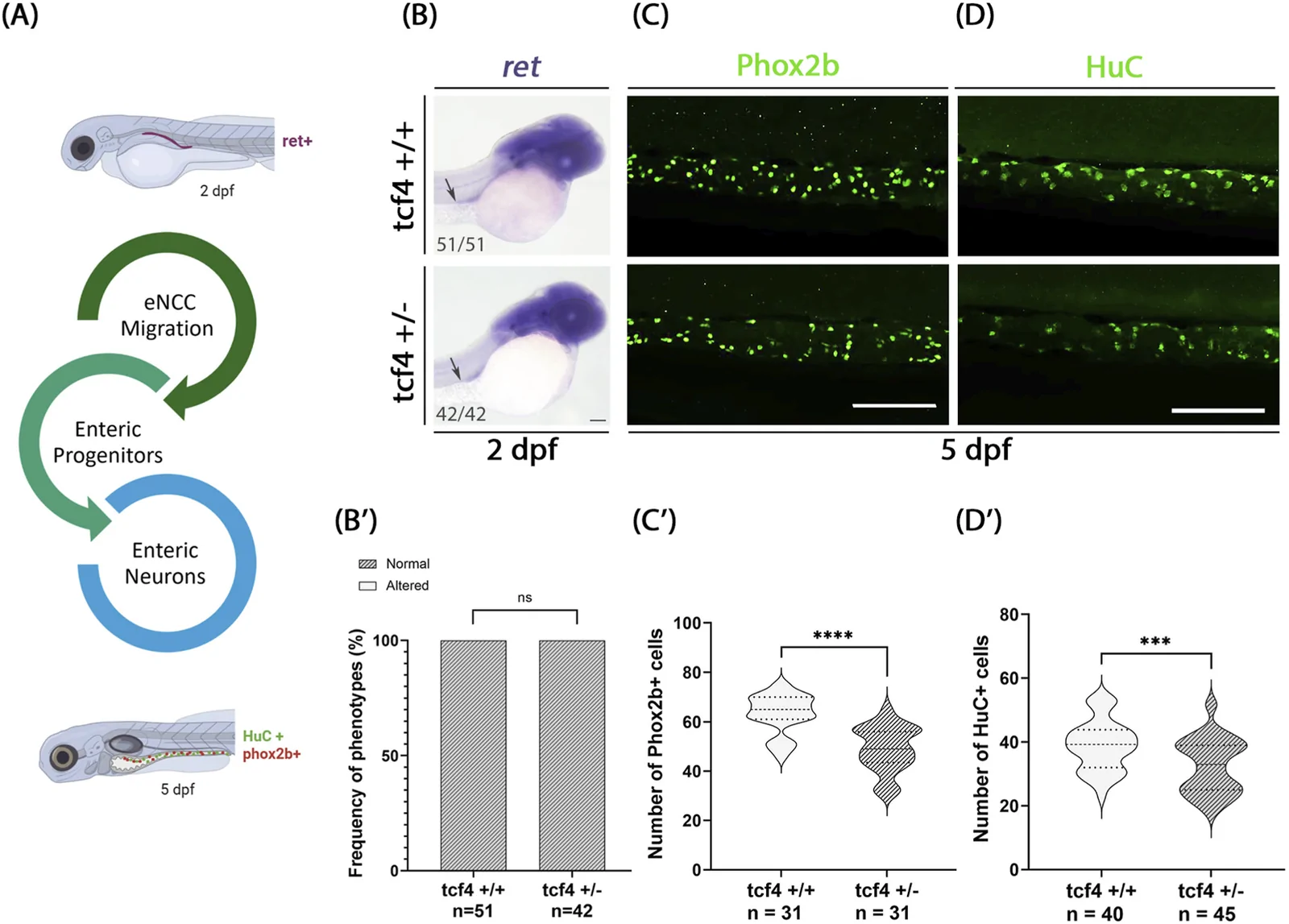
**(A)** Schematic representation of enteric neurons development. At 2 dpf the enteric Neural Crest Cells (eNCC), expressing *ret*, migrate toward the gut. At the level of the gut the enteric progenitors, expressing Phox2b, differentiate into neurons, starting to express HuC. Created with BioRender.com. **(B)** Migrating eNCC marker *ret* show a normal migration toward the gut (arrow) in *tcf4* +/− compared with *tcf4 +/+*. At 5 dpf *tcf4* +/− larvae show a significant reduction of **(C)** enteric progenitors (Phox2b-positive) and **(D)** enteric neurons (HuC-positive), compared with *tcf4 +/+* (B′, C′, D′) Graphic representation of statistical analysis performed, with the relative number of statistical units and level of significance. Fisher’s exact test (right) and Unpaired t-test (left) were used. Scale bar is 100 µm.

**FIGURE 6:**
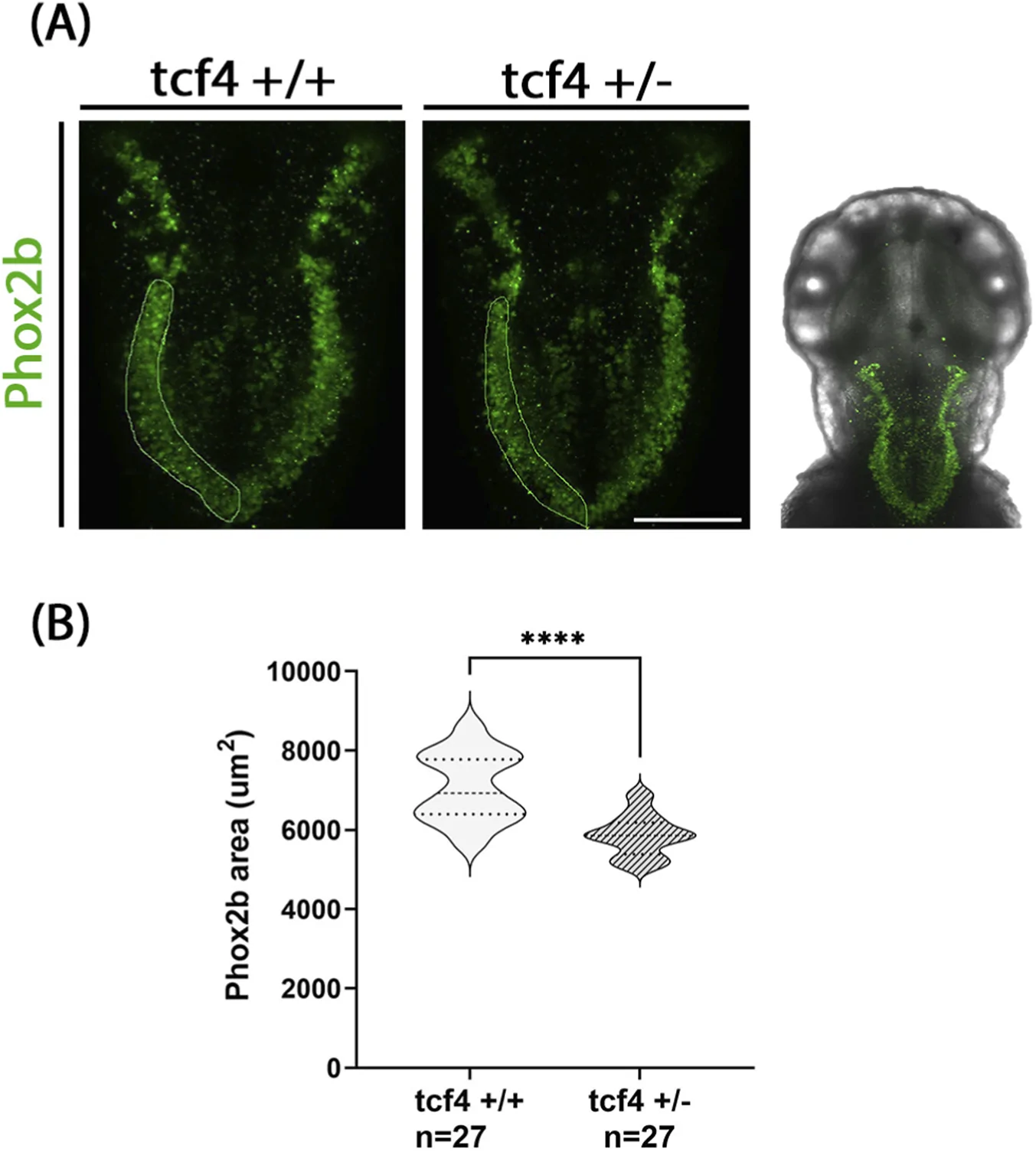
**(A)** In the Central Nervous System, *tcf4* +/− embryo at 3 dpf display a significant reduction of Phox2b-positive area in the hindbrain compared with *tcf4 +/+*. On the right a representative image showing the localization of Phox2b-positive cells in the hindbrain of zebrafish larvae at 3 dpf. **(B)** Graphic representation of statistical analysis performed, showing a significant reduction of Phox2b-positive area, with the relative number of statistical units and level of significance. Unpaired t-test. Scale bar is 100 µm.

To investigate the cellular basis of the observed gastrointestinal defects, we analysed the development of the Enteric Nervous System (ENS) (Figure 5A). To determine whether eNCC migration was affected, we examined the distribution of *ret*-expressing eNCC at 2 dpf (Figure 5B). No significant differences (Figure 5B’; Fisher’s exact test, p-value>0.99, ns) were observed between genotypes, suggesting that initial migration to the gut is preserved and that the requirement for Tcf4 during ENS development becomes critical after the eNCC migration toward the gut. Immunofluorescence analysis at 5 dpf (Figures 5C,D), when eNCC have already colonized the gut and started differentiating into enteric neurons, revealed a significant reduction in the number of Phox2b-positive enteric progenitors (Figure 5C’; unpaired t-test, p-value<0.0001, ****) and HuC-positive enteric neurons (Figure 5D’; unpaired t-test, p-value = 0.0006, ***) in *tcf4* +/− larvae (respectively n = 31, mean = 48.98, SEM = 1.658; n = 45, mean = 32.56, SEM = 1.291) compared with *tcf4 +/+* larvae (respectively n = 31, mean = 63.27, SEM = 1.422; n = 40, mean = 38.95, SEM = 1.245). These results support the hypothesis that an altered development of ENS may underlie the gastrointestinal dysfunction observed in PTHS and in our zebrafish model, shedding light on the molecular and cellular basis of constipation in patients. Of note, analysis of Phox2b immunofluorescence also revealed an altered distribution of Phox2b-positive neurons in the central nervous system (Figure 6A). We observed a significative reduction (Figure 6B; unpaired t-test, p-value<0.0001, ****) in the area of the Phox2b-positive stripe 5 of the inferior dorsal medulla oblongata in *tcf4* +/− zebrafish embryos (n = 27, mean = 5,865, SEM = 99.59) compared with *tcf4 +/+* embryos (n = 27, mean = 7,077, SEM = 167.4) at 3 dpf. These data suggest a shared molecular regulatory network between hindbrain neuronal precursors contributing to the autonomic (visceral) nervous system and NCC precursors contributing to ENS.

### 3.5 Reintroducing human *TCF4* expression rescues the phenotype of *tcf4* +/− larvae

**FIGURE 7:**
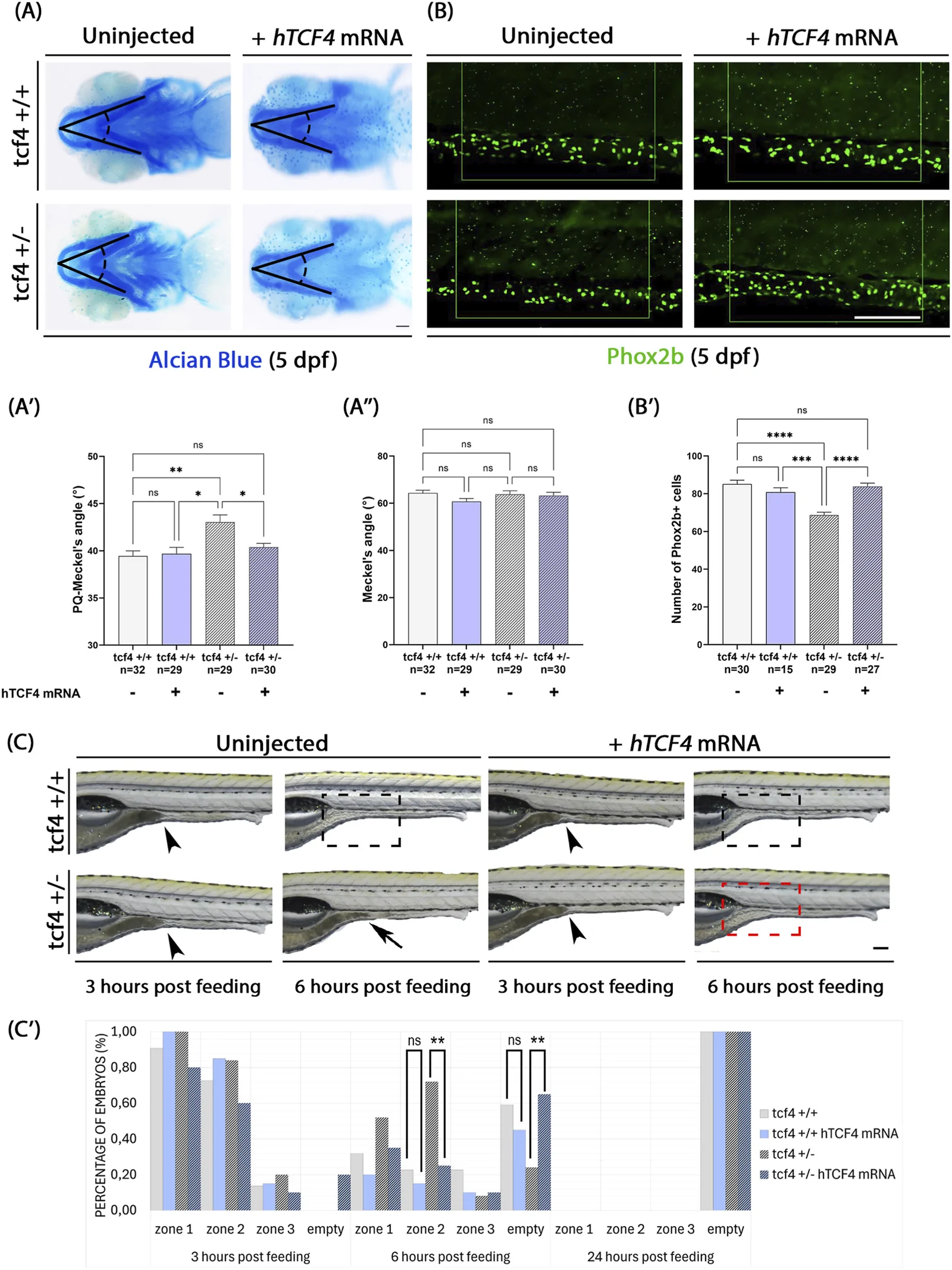
**(A)** Representation of 5 dpf embryos stained with Alcian Blue. The injection of human *TCF4* mRNA in *tcf4* +/− embryos rescued the craniofacial alterations, with a PQ-Meckel’s angle (black angle) significantly different compared to *tcf4* +/− injected embryos and similar to the dimension observed in *tcf4 +/+* larvae (A′), while the Meckel’s angle was not affected (A″). **(B)** Representative images of immunofluorescence performed with Phox2b antibody (B′) *tcf4* +/− larvae injected with *hTCF4* mRNA rescued the number of Phox2b-positive progenitors to the level of *tcf4 +/+* larvae, while the injection of *hTCF4* mRNA in *tcf4 +/+* embryos did not alter the number of enteric progenitors. Respectively, Brown-Forsythe ANOVA test with Dunnet’s T3 for multiple comparisons for PQ-Meckel’s angle, and one-way ANOVA test with Tukey’s test for multiple comparisons, for Meckel’s angle and Phox2b-positive cells. Data are represented as mean ± SEM. Scale bar is 100 µm. **(C)** Representative images of gut transit rescue analysis. At 3 h post feeding all the conditions show the presence of the bolus (arrowhead). However, at 6 h post feeding, while most of the injected and uninjected *tcf4 +/+* had successfully thrived (black dashed box), *tcf4* +/− displayed a delayed gut transit (black arrow), which was recovered in *tcf4* +/− larvae injected with *hTCF4* mRNA (red dashed box). Scale bare is 100 µm (C′) The percentage of injected *tcf4* +/− larvae that emptied the gut and did not retain the bolus in the zone 2 was recovered to the level of *tcf4 +/+* larvae, both injected and uninjected. Uninjected *tcf4 +/+* n = 22, uninjected *tcf4*   n = 25, injected (*hTCF4* mRNA) *tcf4 +/+* n = 20, injected (*hTCF4* mRNA) *tcf4* +/− n = 20. Chi-square test.

To confirm the causal link between *tcf4* LoF and the phenotypes described above, we performed functional rescue experiments by microinjecting human *TCF4* mRNA into *tcf4* +/− zygotes. This approach aims not only to validate our new zebrafish PTHS model but also to highlight the importance of *TCF4* dosage-dependent effects during development. Notably, dose-response experiments in *tcf4 +/+* showed that injection of 400 pg per embryo of *TCF4* mRNA induced craniofacial abnormalities, distinct from *tcf4* haploinsufficiency (Supplementary Figure S1). We observed a TCF4 overexpression specific phenotype showing an alteration of the ethmoidal plate midline chondrification and a peculiar beak-shape with a prominent Meckel’s cartilage. In contrast, a lower dose (200 pg per embryo) did not affect wild-type or *tcf4 +/+* embryos development and was therefore selected for rescue experiments. Regarding the craniofacial phenotype (Figure 7A) observed in *tcf4* +/− larvae, reinstatement of TCF4 expression via human mRNA injection revealed a significant overall effect among groups (Brown-Forsythe ANOVA test, p-value = 0.0003, ***). Subsequent Dunnett’s T3 multiple comparisons analysis showed a significant rescue (Figure 7A’; p-value = 0.0235, *) of PQ-Meckel’s angle in injected *tcf4* +/− larvae (n = 30, mean = 40.38, SEM = 0.4115) compared with uninjected ones (n = 29, mean = 43.03, SEM = 0.7687), reaching values comparable to *tcf4 +/+* larvae, both injected (n = 29, mean = 39.67, SEM = 0.6874) and uninjected (n = 32, mean = 39.44, SEM = 0.5503). Interestingly, the restoration of TCF4 expression in *tcf4* +/− embryos rescued the gastrointestinal phenotype both at the cellular and functional level. First, the number of Phox2b-positive enteric progenitors (Figure 7B) in *tcf4* +/− larvae (injected, n = 27, mean = 83.85, SEM = 1.712; uninjected, n = 29, mean = 68.69, SEM = 1.477) returned to levels comparable to those in *tcf4 +/+* larvae (injected, n = 15, mean = 80.80, SEM = 2.334; uninjected, n = 30, mean = 85.13, SEM = 2.055) at 5 dpf (Figure 7B’; ordinary one-way ANOVA, p-value <0.0001, ****). Moreover, the gut transit (Figure 7C) in *tcf4 +/−* (injected, n = 20; uninjected, n = 25) was recovered (Figure 7C’) to the level of *tcf4 +/+* larvae (injected, n = 20; uninjected, n = 22), including the lack of bolus retention in the rostral intestinal region (zone 2; Chi-square test, p-value = 0.0017, **) and the successful emptying of the gut (Chi-square test, p-value = 0.0057, **). These results confirmed that the human *TCF4* transcript was sufficient to rescue both craniofacial and gastrointestinal phenotypes in heterozygous larvae, without impairing craniofacial structures, Phox2b-positive enteric progenitors’ pool, and gut function when injected into *tcf4 +/+* embryos. Overall, these data indicate that the chosen dosage achieved functional rescue without detectable overexpression-related side effects.

## 4 Discussion

In this study, we established and characterized the first zebrafish model of Pitt-Hopkins Syndrome (PTHS) by generating a *tcf4* knock-out line. This model recapitulates key aspects of the disease leading to the analysis of pathogenetic alterations that are difficult to address in mammalian systems, remaining poorly investigated. The external development, the small dimension and the transparency of zebrafish embryos gave us the possibility to monitor the development of multiple cell types from early phases of development to the terminal differentiation steps. These advantages are particularly relevant when analysing neural crest derivatives. Although specified at the neural plate border, multipotent NCC gain their final fate by integrating multiple signals during migration and within their destination niche (Szabó and Mayor, 2018; Sutton et al., 2021).

The high degree of conservation of the TCF4 bHLH domain sequence, essential for dimerization and DNA-binding, between zebrafish and humans, together with the overlapping expression patterns in the developing CNS and in NCC derivatives, also supports the use of zebrafish as a relevant model to study TCF4 function *in vivo*. In mice, *Tcf4* is required for neuronal differentiation and cortical development, showing a strong end extensive expression in the CNS, especially at level of the cortex and the cerebellum. *Tcf4* loss of function leads to microcephaly, delayed neurogenesis and altered behaviour (Flora et al., 2007; Thaxton et al., 2018; Schoof et al., 2020; Espinoza et al., 2024). Consistently, we observed robust and persistent *tcf4* expression in the developing brain of zebrafish, including forebrain, midbrain, hindbrain, with a strong expression at the level of the cerebellum. *Tcf4* null mice are embryonic or early postnatal lethal (Thaxton et al., 2018), highlighting its essential role during development. Similarly, homozygous *tcf4* knockout in zebrafish severely compromised viability, with only few larvae surviving to adulthood, showing impaired fertility. Facial gestalt is a hallmark of PTHS, with the most frequent manifestation represented by wide mouth and wide nasal bridge (Zollino et al., 2019). In zebrafish, we observed an increase in the PQ-Meckel’s angle, resulting in a widening of the jaw, recalling the most distinctive traits of PTHS craniofacial dysmorphism. Despite evolutionary differences between zebrafish and humans’ craniofacial morphogenesis, the widening of the Meckel’s articulation with the palatoquadrate determined by *tcf4* haploinsufficiency in zebrafish suggest a critical role of *tcf4* for proper craniofacial morphogenesis. Furthermore, the craniofacial structures originate largely from cranial NCC, prompting the hypothesis of *tcf4* role during NCC derivatives development. These data shed a light on unexplored aspects of PTHS, laying the groundwork for future studies to unveil the role of *tcf4* in NCC development. Another common yet understudied aspect of PTHS is gastrointestinal (GI) complications, with frequent severe chronic constipation. Patients show a reduced bowel transit, leading to intestinal pseudo-obstruction. Those symptoms have a strong impact on the quality of life of patients and their families (Goodspeed et al., 2018; Zollino et al., 2019; Comisi et al., 2023) leading, in few cases, to death (Koppen et al., 2023). Moreover, PTHS patients have speech difficulties and are often non-verbal, making it more challenging for caregivers to quickly identify the origin of their pain. Indeed, severe abdominal bloating and intestinal obstruction strongly impair the everyday life of patients, leading to persistent agitation and severe abdominal pain (Comisi et al., 2023). The cellular basis underlying these life-long functional impairments has not yet been identified. Zebrafish have been shown to be a good model for gastrointestinal diseases due to their small size and transparency of the larvae (Doodnath et al., 2010). The gut functionality mainly depends on the regulatory role of the enteric nervous system, which innervates and coordinates the smooth muscle contractions. The ENS of zebrafish is similar to the mammalian one, although the reduced architectural complexity, consisting of a single layer of cells scattered or organized in small cluster between the circumferential and longitudinal muscle layer (Ganz, 2018). Furthermore, the processes directing gastrointestinal development are conserved from fish to mammals. Enteric neurons originate from a subpopulation of enteric neural crest cells (eNCCs) expressing Phox2b. Those cells, starting from 32 hpf, detach from the closing hindbrain vesicle and migrate medially and ventrally toward the developing gut. Within 3 dpf they have colonized the distal region of the hindgut and start differentiating in post-mitotic neurons by expressing the pan-neuronal marker HuC/HuD. Up to 7 dpf enteric neurons differentiate into different neuronal subtypes, such as cholinergic and serotoninergic neurons, that regulate the peristaltic movements necessary for digestive tract functioning (Doodnath et al., 2010; Ganz, 2018; Moreno-Campos et al., 2024; Uribe, 2024; Rogers et al., 2025). Of note, recent high-throughput single-cell RNAseq analysis during development identified an expression of *tcf4* both in NCC, including pharyngeal arches and enteric NCC, between 18 and 120 hpf, and in enteric neurons, between 40 and 120 hpf (Sur et al., 2023). Although impaired upper gastrointestinal transit has been reported in PTHS mouse models (Grubišić et al., 2015), the cellular basis of this phenotype has remained unclear. The evaluation of gut functionality confirmed the impairment observed in mice, with *tcf4* +/− showing a reduced spontaneous peristalsis frequency, that reflected in an altered swallow-induced gut transit, mimicking the constipation observed in human patients. To deepen our knowledge on the cellular basis for GI issues, we hypothesize a correlation between functional observation with the significant reduction in Phox2b-positive enteric progenitors and HuC-positive enteric neurons observed in *tcf4* +/− larvae compared with *tcf4 +/+*. Further strengthening this hypothesis, the expression of *hTCF4* mRNA in *tcf4* +/− larvae rescued both the reduction of the Phox2b-positive enteric progenitors and the delayed gut transit, suggesting that the gastrointestinal issues observed in PTHS patients could be related to an altered ENS development. Given that enteric neurons derive from enteric NCCs, these data further support a role for Tcf4 in NCC-derived lineages. Previous studies hypothesized that an impairment of ENS development could led to the GI issues observed in PTHS patients (Amiel et al., 2007; Zollino et al., 2019; Zweier et al., 2007). This is the first time this hypothesis has been tested and investigated in an *in vivo* animal model, shedding light on the requirement of *tcf4* in ENS development.

The general dysautonomia observed in patients also involves respiratory control. Breathing pattern abnormalities of PTHS patients feature intermittent hyperventilation followed by episodes of apnea, usually of brief duration. TCF4 interacts with the proneural transcription factor ASCL1. It has been hypothesized that breathing and gastrointestinal defects of PTHS patients could be related to an altered interaction of defective TCF4 with the ASCL1-PHOX-RET pathway (Zweier et al., 2007; De Pontual et al., 2009). Medullar Phox2b-positive parafacial neurons of the retrotrapezoid nucleus, a respiratory central control region, resulted depleted in a PTHS mouse model characterized by a truncated *Tcf4* (Cleary et al., 2021). It is worth noting that variants of uncertain significance in *ASCL1* have been reported in patients presenting with clinical features overlapping with Congenital Central Hypoventilation Syndrome (CCHS, OMIM: #209880), with or without Hirschsprung disease (HSCR) (Malbos et al., 2024). CCHS is primarily caused by pathogenic variants in *PHOX2B* and is characterized by impaired development of noradrenergic neurons, leading to abnormal autonomic control of breathing. In contrast, the most common genetic cause of HSCR is represented by pathogenic variants in *RET*, resulting in aganglionosis of the myenteric plexus of the gastrointestinal tract (Peippo et al., 2006). However, to date, only one PTHS patient has been diagnosed with HSCR disease (Peippo et al., 2006; Goodspeed et al., 2018). Moreover, the GI defects observed in PTHS are generally less severe than those seen in HSCR, in which the absence of enteric ganglion cells in the affected intestinal segment leads to functional obstruction and colonic dilatation, also referred to as megacolon. These differences strongly correlate with our observation of a partial loss of enteric neurons and progenitors, rather than a complete aganglionosis observed in HRSC patients. In line with this observation, we did not detect any alterations in the *ret* expression during eNCC migration toward the gut in *tcf4* heterozygous larvae, compared with *tcf4 +/+* animals. These data, together with the functional alterations shown by our model and mouse models of PTHS, point to an altered development of the enteric progenitor’s pool, which determines a significant reduction in the enteric neurons and, consequently, an altered control of peristaltic gut movement. Our data demonstrates an impairment of Phox2b-positive neuronal lineages development both in the CNS and in the ENS support the possible involvement of the ASCL1-PHOX pathway in the pathogenesis of GI defects observed in PTHS patients, adding the fundamental contribution of TCF4 as a key player in the orchestration of CNS and ENS development. Although our data demonstrate a significant reduction in Phox2b-positive enteric progenitors and differentiated enteric neurons in *tcf4* haploinsufficient embryos, the molecular mechanisms underlying these defects remain to be elucidated. As a basic helix-loop-helix transcription factor, TCF4 may directly or indirectly regulate the transcriptional programs required for Enteric Nervous System development. Whether *PHOX2B* represents a direct transcriptional target of TCF4, or the observed changes arise through indirect regulatory pathways, remains an open question. Future studies employing approaches such as RNA sequencing, chromatin immunoprecipitation (ChIP) or transcriptional reporter assays will be valuable to define the regulatory relationship between *TCF4* and *PHOX2B* and in further clarify the gene regulatory networks disrupted in PTHS. Moreover, the identification of altered ENS development as the cause of the GI issues in PTHS patients could lay the basis for the application of cellular-based regenerative therapeutic approaches that are currently under investigation for Hirschsprung disease (Alhawaj, 2022; Yoshimaru et al., 2024). These therapeutic strategies could significantly improve the quality of life of patients and their families and show a high applicative potential for the near future. The present study shows that the craniofacial and gastrointestinal alterations of PTHS patients could have a common developmental origin, highlighting the role of Tcf4 in NCC. TCF4 haploinsufficiency underpinnings PTHS implies a therapeutic potential, suggesting that the restoration of TCF4 function could serve as a therapeutic intervention for PTHS. This hypothesis has been strengthened by findings obtained on PTHS-patients derived brain organoids, that could overcome the transcriptional and cytoarchitectural deficits of *TCF4* LoF, if its expression is reinstated (Papes et al., 2022). Moreover, the *in vivo* mouse models of PTHS are being examined to evaluate the optimal window for genetic intervention. As a matter of fact, a conditional early embryonic TCF4 restoration of a PTHS mice model showed a complete rescue of locomotory, learning and memory behaviour (Kim et al., 2022). Corroborating these observations, we showed that the craniofacial and gastrointestinal alterations of *tcf4* +/− embryos can be completely rescued by the human *TCF4* mRNA, strongly supporting not only the specificity of the phenotype, but also representing a novel preliminary proof of concept of a possible therapeutic strategy aimed to restore enteric neurons development. Thus, our analysis bolsters the hypothesis that molecular, functional and morphological rescue of PTHS defects is possible when Tcf4 restoration is performed early in development. However, embryonic interventions are unlikely to be performed in human patients. Recent studies have shown that postnatal reinstatement of TCF4 expression in neurons of PTHS mouse model could partially recover some behavioural defects if the treatment is performed at P1 stage. However, the restoration of TCF4 at P14, roughly corresponding to the toddler stage in human, could only recover the object location memory, depending on the hippocampus control. Interestingly, this region is highly plastic and retains high TCF4 expression also in the adult mouse brain, suggesting that a later therapeutic intervention could be successful if delivered to highly plastic tissues (James et al., 2025). Finally, the unique opportunity to perform gene gain and loss of function experiments in zebrafish embryos allowed us to draw attention on the TCF4 dosage dependent effect that has never been evaluated so far. This is a key information to be considered to avoid over-expression toxicity in gene therapy approaches. We showed that the human *TCF4* mRNA overexpression alters craniofacial development in zebrafish embryos. Thus, TCF4 reinstatement dosage should be evaluated for target cell types to avoid a gain of function phenotype. In conclusion, even if a gene therapy clinical trial targeting brain cells is undergoing (ClinicalTrials.gov ID: NCT07135050 2025–2029) further research is needed to propose therapeutical approaches aimed to recover tissue-specific phenotypes in specific time windows. In this perspective, the availability of a new *in vivo* model that enables live imaging and the simultaneous study of multiple TCF4-expressing tissues alterations could provide a comprehensive understanding of its functions, enabling the investigation of understudied aspects of the disease. The external development of zebrafish will also contribute to the determination of the time-window requirement of Tcf4 in different tissues representing a strategic *in vivo* platform for drug screening and to evaluate proof of concept interventions based on RNA or gene therapy approaches.

## Data Availability

The raw data supporting the conclusions of this article will be made available by the authors, without undue reservation.

## In memoriam

This manuscript is dedicated to the beloved memory of Claudio Ori.
