# Supplementary material for: TCF4 at the crest of development: a zebrafish model to explore craniofacial and gastrointestinal defects in Pitt-Hopkins syndrome
Source: Front Cell Dev Biol. 2026 Sep 4;14:1907674. doi: 10.3389/fcell.2026.1907674 (PMC13585727; doi:10.3389/fcell.2026.1907674)
Supplement: Supplementary file 1 [file DataSheet1.pdf]

Supplementary Figure

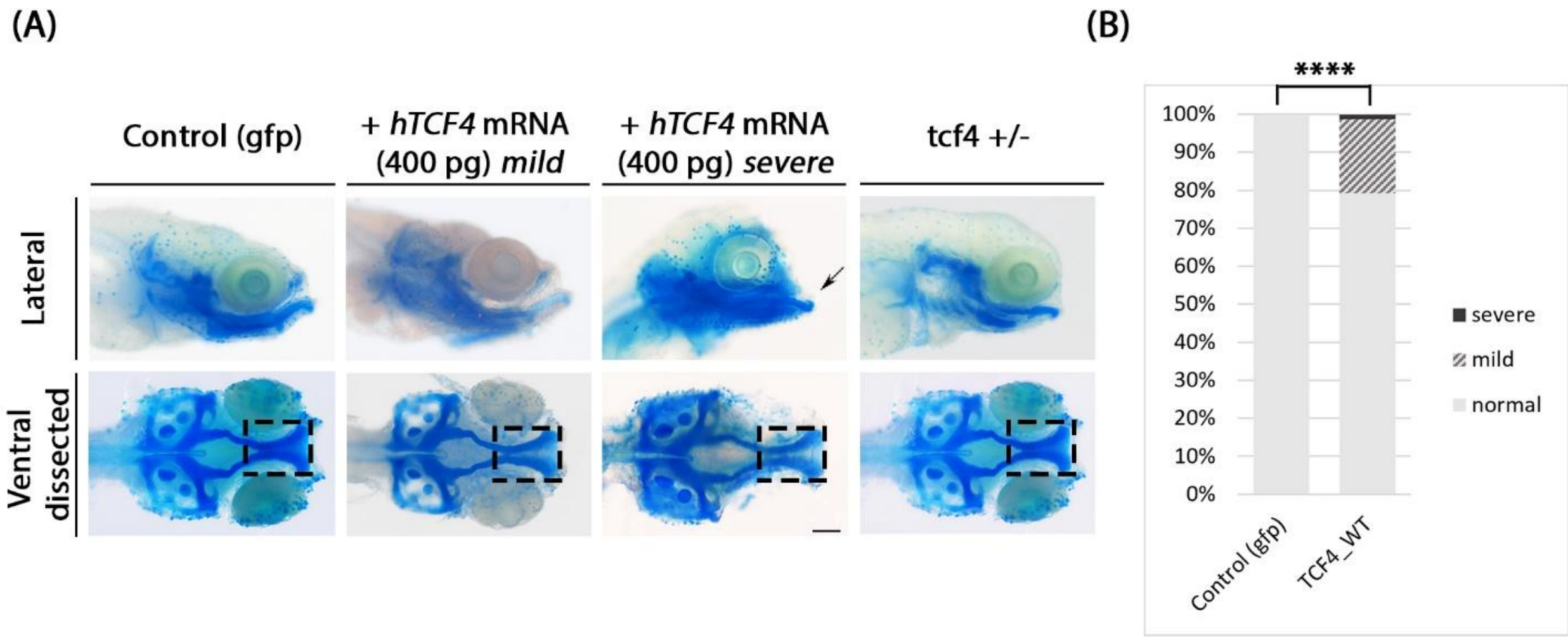

(C)

	Normal	Mild	Severe	Fisher's exact test
**Control (gfp)**	165	0	0	p-value < 0.0001, ****
**hTCF4 mRNA (400 pg)**	122	30	2	

Figure S1 (A) Representation of 5 dpf embryos stained with Alcian Blue. The injection of human *TCF4* mRNA at 400 pg per embryo showed craniofacial alteration classified as mild, showing altered chondrification of the ethmoid plate midline (dashed box), and severe, with a prominent jaw (arrow) and profoundly altered morphogenesis of the ethmoidal plate (dashed box). The craniofacial defects observed with the overexpression of human *TCF4* were strikingly different from the ones observed with *tcf4* loss of function (tcf4 +/-). In particular, the altered morphogenesis of the ethmoidal plate seems to be specifically induced by the overexpression of human *TCF4* and was not observed in tcf4 +/- larvae. (B) Percentages of phenotypes classified as normal, mild and severe, with the relative level of significance. (C) Number of statistical units and level of significance. Fisher's exact test. Scale bar is 100 µm.

Supplementary Data

Script for the gut motility analysis to be performed in MATLAB software (https://it.mathworks.com/products/matlab.html?s_tid=m_footer_matlab).

```
%% GUT MOTILITY ANALYSIS

clc; clear; close all;

%% Constant parameters

pixelPerMillimeterBase = 226.5; % 1mm at 1X magnification corresponds to 226.5 pixels

magnificationScale = 10;  % Microscope magnification
```

```
pixelsPerMillimeter = pixelPerMillimeterBase * magnificationScale;

pixelsPerMicrometer = pixelsPerMillimeter / 1000;  % Convert px/mm to px/µm

pixelSizeMicrometers = 1 / pixelsPerMicrometer;  % µm/px

numSomites = 3; % Number of somites to set the fixed line length

frameStep = 2;  % Process every 2 frames to speed up analysis (adjust as needed)

%% === 0. LOAD INITIAL DATA ===

choice = LoadInitialData();

% If the user chose to open a new video

if strcmp(choice, 'new')

   %% === 1. SELECT THE VIDEO ===

   [videoReader, numFrames, fileName, filePath] = SelectVideo();

   %% === 2. OPEN REFERENCE FRAME AND POSITION A FIXED LINE WITH PREDEFINED LENGTH ===

   [lineXCoordinates, lineYCoordinates, numPoints] = SetFixedLinePosition(videoReader, pixelsPerMillimeter, numSomites);

   %% === 3. INTERPOLATE PIXEL INTENSITY ALONG THE LINE ===

   [intensityMatrix, frameIndex] = InterpolateLineIntensity(videoReader, numFrames, lineXCoordinates, lineYCoordinates, numPoints, frameStep);

   % === Seconds for the y axis (time) ===

   effectiveFps = videoReader.FrameRate / frameStep;

   timeVector = (0:frameIndex-1) / effectiveFps;

   %% === 4. SAVE DATA FOR FUTURE ANALYSIS ===

   SaveInterpolatedData(intensityMatrix, timeVector, numPoints, fileName, filePath);

   %% === 5. POST PROCESSING OF THE MATRIX (INTERPOLATED DATA) ===

   [filteredMatrix] = FilterIntensityMatrix(intensityMatrix, effectiveFps);

   % === Matrix conversion into grayscale ===

   filteredMatrixGrayscale = mat2gray(filteredMatrix);

   %% === 6. KYMOGRAPH GENERATION ===

   totalLengthMicrometers = numPoints * pixelSizeMicrometers;
```

```
    [selectedImage] = GenerateKymograph(filteredMatrixGrayscale, timeVector, totalLengthMicrometers, fileName, filePath);

% If user chose to load saved data

elseif strcmp(choice, 'saved')

    %% === 6a. LOADING PREVIOUSLY SAVED DATA FOR KYMOGRAPH ANALYSIS ===

    [intensityMatrix, timeVector, numPoints] = LoadSavedData()

    %% === 6b. POST PROCESSING OF THE MATRIX (INTERPOLATED DATA) ===

    filteredMatrix = FilterIntensityMatrix(intensityMatrix, 1/(timeVector(2)-timeVector(1)));

    filteredMatrixGrayscale = mat2gray(filteredMatrix);

    %% === 6c. KYMOGRAPH GENERATION ===

    totalLengthMicrometers = numPoints * pixelSizeMicrometers;

    % Image files are not saved again when loading from saved data

    [selectedImage] = GenerateKymograph(filteredMatrixGrayscale, timeVector, totalLengthMicrometers, '', '');

else

    error('Choice not recognized. Please try again.');

end

%% === 7. AVERAGE VELOCITY CALCULATION ===

CalculateVelocity(selectedImage, timeVector, totalLengthMicrometers);

%% === 8. AVERAGE FREQUENCY AND INTERVAL CALCULATION ===

CalculateFrequency(selectedImage, timeVector, totalLengthMicrometers);

%% === 9. AVERAGE AMPLITUDE CALCULATION OF PERISTALTIC WAVES ===

CalculateAmplitude(selectedImage, timeVector, totalLengthMicrometers);
```
